# Newspapers

**Published:** 1879-05

**Authors:** 


Perhaps there is no other country in the world in which light literature is so much encouraged as it is in this. In New York and Boston alone, there are more weekly publications, devoted entirely to productions of this class, than in the whole of the British Empire. Yet all of these publications not only live, but enjoy a wider patronage and a more extensive circulation than the best news-journals of either city can boast of, and should be realizing 'rapid fortunes' for their proprietors, especially as all the half-fledged litterateurs of the day—love-sick young ladies and misanthropical young gentlemen—supply them gratuitously with their lucubrations. What woman or child, capable of reading, is there in this city, who is not accustomed to purchase or borrow some of these papers, and devour their contents with eager interest? Yet what are all these publications made up of, or what is there in them, either in the way of matter or of style, that can entitle them to perusal? Little or nothing; love-stories—love-stories as like each other in incident, style, and denouement as if they were all cut out of the same cloth, as well as made after the same fashion; tales of beautiful blondes, with meek blue eyes, and haughty brunettes, with flashing black ones; and lovers who are all heroes, and poets, and extraordinary genuises.

"Away with all such trash as this, we say! Publications of this kind never did, and never will, answer any useful purpose whatever. On the other hand, they are absolutely injurious in their tendencies; they serve to give those who are addicted to their perusal false views of life—to unsettle the balance of their whole minds, and make them visionary dreamers, instead of earnest doers, in the world.

"The same remarks will also apply to most of the cheap novels of the day, and to the whole of that 'yellow-covered literature' which was of late the rage, though it was the joint product of bile and delirium; and we would scarcely regret it, if all the prose fictions that ever were written were condemned in a lump to the same fate to which the favorite authors of Don Quixote were consigned, for very few of them teach any useful lessons, or contain anything which might not as well go unlearned altogether, or be forgotten as speedily as possible.

"At the best, all fiction, whether prose or verse, is but a luxury of the mind, is unfit for its daily food, and cannot support it in health and vigor. We cannot live on sweatmeats, and would soon die, were we confined to ice-cream and pastries. No father or guardian should ever allow those under his charge to indulge in the habitual reading of 'tales,' if he has any regard for their intellectual or moral health.

